# Two- and Three-Dimensional Echocardiography in the Evaluation of the Paravalvular Regurgitation of the Mitral Valve Annuloplasty Ring

**Published:** 2018-10

**Authors:** Ahmet Guner, Gokhan Kahveci

**Affiliations:** *Department of Cardiology, Kosuyolu Kartal Training and Research Hospital, Istanbul, Turkey.*

**Keywords:** *Mitral valve annuloplasty*, *Echocardiography*, *Echocardiography, Doppler*, *Echocardiography, three-dimensional*

A 20-year-old male patient was admitted to our emergency department with the complaints of cough and dyspnea. He had undergone atrial septal defect repair and mitral annuloplasty ring (St. Jude No.34) placement due to severe mitral regurgitation 5 years earlier. Physical examination revealed blood pressure of 175/110 mm Hg with tachypnea (24/min.), apical holosystolic murmur, and elevated jugular venous pressure. Electrocardiography showed atrial fibrillation and a mean heart rate of 125 bpm. After initial medical stabilization, bedside transthoracic echocardiography was performed, which revealed left ventricular systolic dysfunction with an estimated ejection fraction of 45%, mildly dilated left ventricle with global wall hypokinesis, severe mitral regurgitation due to mitral ring dehiscence, para-ring leak with a centrally directed jet, and moderate tricuspid regurgitation (Figure 1 and Figure 2). For further evaluation, transesophageal echocardiography (TEE) was performed. The mitral ring was visible above the anterior part of the annulus at 0o angle. Color Doppler flow demonstrated significant paravalvular regurgitation between the ring and the mitral annulus, with a jet directed through the para-ring to the roof of the left atrium (Figure 3) and another jet slightly toward the posterior wall of the left atrium. Three-dimensional TEE confirmed the severe para-ring leak of the anterior mitral annulus toward the anteromedial segment and also demonstrated an anterior mitral annulus para-ring hole in the anteromedial segment (Figure 4). The patient was scheduled for reintervention (surgical or percutaneous) on January 12, 2018, but he refused. From then until the presenting this case, he had received medical therapy for 5 months.

Mitral valve annuloplasty may be complicated by mitral regurgitation from a dehisced rocking ring. Two-dimensional echocardiography still plays a role in the initial assessment in that it raises suspicion that there might be an anomaly associated with the existence of a significant paravalvuler reflux in the presence of para-ring leak. The case presented herein underscores the significant role of three-dimensional TEE in the assessment of mitral valve dehiscence and para-ring leak.

**Figure 1:**
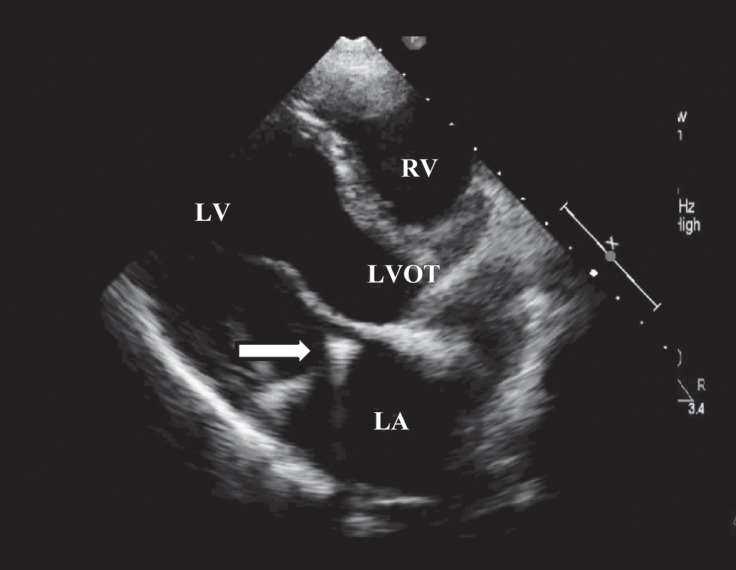
Parasternal long-axis view of the echocardiogram, showing gross dehiscence in the mitral ring (arrow).

**Figure 2:**
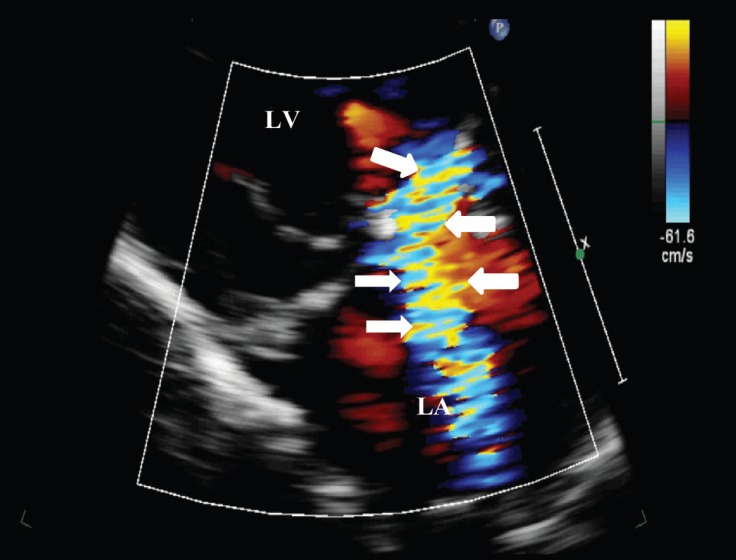
Transthoracic echocardiography view of the centrally directed mitral regurgitation jet by color Doppler (arrows).

**Figure 3:**
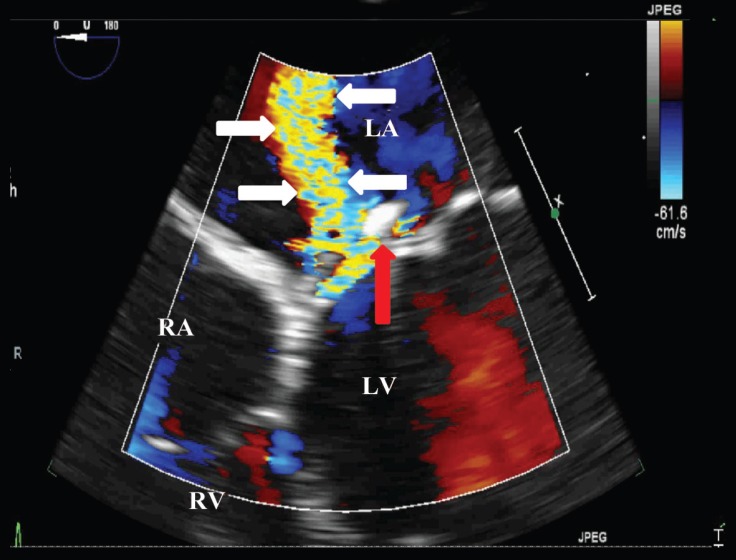
Transesophageal echocardiogram (4-chamber view), showing severe mitral regurgitation (white arrows) and another jet slightly toward the posterior wall of the left atrium (red arrow).

**Figure 4:**
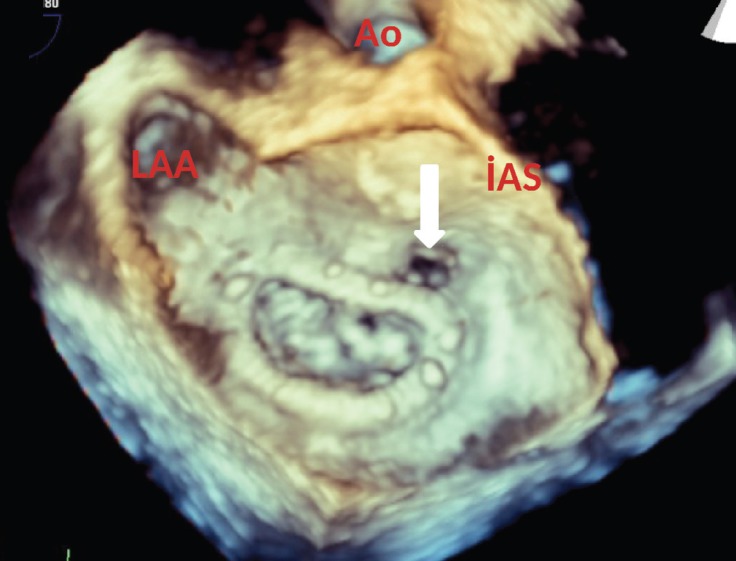
Three-dimensional transesophageal echocardiography, showing the anterior mitral annulus para-ring hole in the anteromedial segment (arrow).

## Notes:

***This paper should be cited as: ***Guner A, Kahveci G. Two- and Three-Dimensional Echocardiography in the Evaluation of the Paravalvular Regurgitation of the Mitral Valve Annuloplasty Ring. J Teh Univ Heart Ctr 2018;13(4):191-192.

